# Author Correction: Shifts in evolutionary lability underlie independent gains and losses of root-nodule symbiosis in a single clade of plants

**DOI:** 10.1038/s41467-024-50907-8

**Published:** 2024-08-01

**Authors:** Heather R. Kates, Brian C. O’Meara, Raphael LaFrance, Gregory W. Stull, Euan K. James, Shui-Yin Liu, Qin Tian, Ting-Shuang Yi, Daniel Conde, Matias Kirst, Jean-Michel Ané, Douglas E. Soltis, Robert P. Guralnick, Pamela S. Soltis, Ryan A. Folk

**Affiliations:** 1 Florida Museum of Natural History, University of Florida, Gainesville, FL USA; 2 Department of Ecology and Evolutionary Biology, University of Tennessee, Knoxville, TN 37996-1610 USA; 3 Germplasm Bank of Wild Species, Kunming Institute of Botany, Chinese Academy of Sciences, Kunming, 650201 Yunnan China; 4 The James Hutton Institute, Invergowrie Dundee, Scotland UK; 5 Centro de Biotecnología y Genómica de Plantas (CBGP), Universidad Politécnica de Madrid (UPM)-Instituto Nacional de Investigación y Tecnología Agraria y Alimentaria (INIA-CSIC), Campus de Montegancedo, Pozuelo de Alarcón, Madrid, 28223 Spain; 6 Genetics Institute, University of Florida, Gainesville, FL USA; 7 School of Forest, Fisheries and Geomatic Sciences, University of Florida, Gainesville, FL USA; 8 Department of Bacteriology, University of Wisconsin-Madison, Madison, WI 53706 USA; 9 Department of Agronomy, University of Wisconsin-Madison, Madison, WI 53706 USA; 10 Biodiversity Institute, University of Florida, Gainesville, FL USA; 11 Department of Biology, University of Florida, Gainesville, FL USA; 12 Department of Biological Sciences, Mississippi State University, Mississippi State, MS USA

**Keywords:** Phylogenetics, Rhizobial symbiosis, Plant evolution

Correction to: *Nature Communications* 10.1038/s41467-024-48036-3, published online 27 May 2024

In this article the funding from the ‘National Science Foundation of China (No. 31720103903)’ was omitted. The original article has been corrected.

