# Supplementary material for: Shifts in evolutionary lability underlie independent gains and losses of root-nodule symbiosis in a single clade of plants
Source: Nat Commun. 2024 May 27;15:4262. doi: 10.1038/s41467-024-48036-3 (PMC11130336; doi:10.1038/s41467-024-48036-3)
Supplement: Supplementary file 1 — Supplementary Information [file 41467_2024_48036_MOESM1_ESM.pdf]

Supplementary Table 1. Summary of taxonomic sampling of the NFC. Number of genera and species assignment to genera is based on The Plant List (version 1.1; http://www.theplantlist.org) except for Leguminosae subfamilies, which follow LPWG (2017).

Order	Family or Leguminosae Subfamily (Fabales)	Number of samples	Genera sampled/Total genera
Fabales	Papilionoideae	5121	443/503
Fabales	Caesalpinioideae	2100	146/148
Fabales	Dialioideae	38	16/17
Fabales	Detarioideae	367	72/84
Fabales	Cercidoideae	161	9/12
Fabales	Duparquetioideae	1	1/1
Fabales	Polygalaceae	320	22/27
Fabales	Surianaceae	3	4/4
Fabales	Quillajaceae	2	1/1
Rosales	Rosaceae	1917	99/109
Rosales	Cannabaceae	70	9/9
Rosales	Rhamnaceae	558	51/57
Rosales	Moraceae	698	44/49
Rosales	Elaeagnaceae	63	3/4
Rosales	Ulmaceae	41	7/8
Rosales	Urticaceae	642	50/58
Rosales	Dirachmaceae	1	1/1
Rosales	Barbeyaceae	1	1/1
Fagales	Betulaceae	92	6/6
Fagales	Casuarinaceae	37	4/4

Fagales	Nothofagaceae	12	1/1
Fagales	Juglandaceae	75	11/13
Fagales	Ticodendraceae	2	1/1
Fagales	Fagaceae	417	7/9
Fagales	Myricaceae	12	4/5
Cucurbitales	Datiscaceae	2	1/1
Cucurbitales	Apodanthaceae	3	3/3
Cucurbitales	Anisophylleaceae	5	2/4
Cucurbitales	Corynocarpaceae	2	1/1
Cucurbitales	Coriariaceae	5	1/1
Cucurbitales	Cucurbitaceae	343	95/134
Cucurbitales	Tetramelaceae	1	2/2
Cucurbitales	Begoniaceae	197	2/2

Supplementary Table 2. Results of model fit tests for the three analyses (A-C). The best model for each analysis is highlighted in green.

A. Results of model fit test for the main analysis testing one to five hidden rate categories. (The model-fit of the two-state three-rate model is reported here.)

Number of Rate Categories	"Precursor" state?	AICc	deltaAIC
1	NA	466.491	53.61
2	No	415.33	2.443
2	Yes	413.95	1.063
2 ; 3 state	Yes	462.063	49.176
3	No	415.074	2.187
3	Yes	412.887	0
4	No	426.421	13.534
4	Yes	451.759	38.872
5	No	433.859	20.972
5	Yes	453.143	40.256

B. Results of model fit tests for fixed-ancestral-state analysis.

Number of Rate Categories	NFC Ancestor	"Precursor" state?	logLik	AICc	deltaAIC
1	RNS-present	NA	-249.007	502.014	83.244
2	RNS-present	No	-209.77	431.555	12.785
2	RNS-present	Yes	-211.37	432.751	13.981
3	RNS-present	No	-202.302	428.605	9.835
3	RNS-present	Yes	-211.867	418.77	0
3	RNS-absent	Yes	-195.457	412.91	NA

4	RNS-present	No	-211.070	438.655	19.885
4	RNS-present	Yes	-210.970	450.555	31.785
5	RNS-present	No	-212.033	472.210	53.44
5	RNS-present	Yes	-213.012	481.112	62.34

C. Results of model fit tests for alternative topology

Number of Rate Categories	"Precursor" state?	AICc	deltaAIC
1	NA	465.996	51.615
2	No	418.746	4.365
2	Yes	416.748	2.367
3	No	416.038	1.657
3	Yes	414.381	0
4	No	424.814	10.433
4	Yes	424.437	10.056
5	No	434.914	20.553
5	Yes	428.137	13.756

Supplementary Table 3. Confidence intervals for transition rates estimated under the best model for the main topology (Fig. 1). Note that rows 6-11 are identical to rows 12-17 because, under an ARD HRM model as implemented in corHMM, transitions between rate categories must be equal across observed states.

Transition	Description	Rate Category	ML rate	Confidence Interval
**(2,R1) -> (1,R1)**	RNS-Loss	Intermediary hidden state	0.0000	(0, 4.290e-09)
**(1,R1) -> (2,R1)**	RNS-Gain	Intermediary hidden state	0.0977	(0.032,0.180)
**(2,R2) -> (1,R2)**	RNS-Loss	Precursor	1.6373	(0.718,6.846)
**(1,R2) -> (2,R2)**	RNS-Gain	Precursor	0.0000	(0,9.942e-09)
**(2,R3) -> (1,R3)**	RNS-Loss	Non-precursor (absorbing)	0.0006	(0.00047,0.001)
**(1,R2) -> (1,R1)**	Gain of intermediary hidden state	NA	0.0086	(0.007,0.020)
**(1,R3) -> (1,R1)**	None	NA	0.0000	(0,6.928e-09)
**(1,R1) -> (1,R2)**	Loss of intermediary hidden state to precursor	NA	0.0180	(0.014, 0.042)
**(1,R3) -> (1,R2)**	None	NA	0.0000	(0,2.674734e-09)
**(1,R1) -> (1,R3)**	Loss of intermediary hidden state to absorbing non-precursor state	NA	0.1116	(0.078,0.200)
**(1,R2) -> (1,R3)**	Loss of precursor to absorbing non-precursor state	NA	0.0271	(0.017,0.037)
**(2,R2) -> (2,R1)**	None	NA	0.0086	(0.007,0.020)
**(2,R3) -> (2,R1)**	None	NA	0.0000	(0,6.928e-09)

**(2,R1) -> (2,R2)**	None	NA	0.0180	(0.014, 0.042)
**(2,R3) -> (2,R2)**	None	NA	0.0000	(0,2.674734e-09)
**(2,R1) -> (2,R3)**	None	NA	0.1116	(0.078,0.200)
**(2,R2) -> (2,R3)**	None	NA	0.0271	(0.017,0.037)

Supplementary Figure 1. Supplementary phylogenetic tree. Zoomable phylogeny presented in Fig. 1 with branches colored by all six inferred hidden state (ancestral RNS status + rate category). For full explanation of colors and rate categories, see Supplementary Fig. 13: RNS+ =blue (“stable”), purple (no name), and green (“rapid-loss”); RNS-absent=orange (“precursor”), red (“intermediary hidden-state”, and gray (“non-precursor").

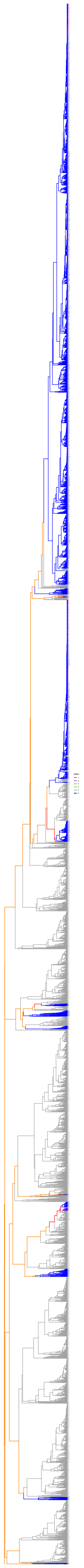
states
1
2
3
4
5
6

Supplementary Figure 2. Results of transition rate estimation and ancestral character state reconstruction based on an alternative NFC backbone topology (see Supplementary Note 4). A. Character states inferred by joint estimation of ancestral character states. Non-NFC clades are scaled down (unlabeled section) to highlight the NFC. NFC orders and legume subfamilies are indicated by colored bars. Branches are colored by the state estimated at their tipward node. B. Transition rate matrix of estimated rates from states listed on left to states listed at top. Rows are colored to correspond to colors in panel A.

A

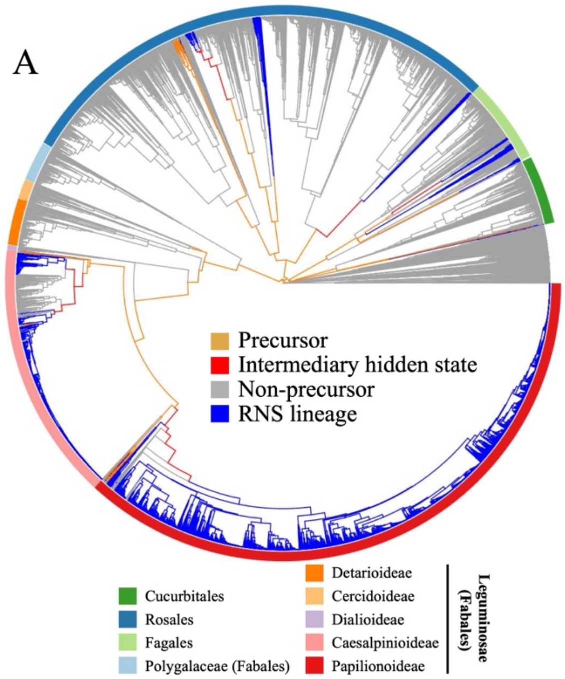

B

	(1,R1)	(2,R1)	(1,R2)	(2,R2)	(1,R3)	(2,R3)
(1,R1)	-	.0977	.0180	NA	.1116	NA
(2,R1)	.0000	-	NA	.018	NA	.1116
(1,R2)	.0086	NA	-	.000	.0271	NA
(2,R2)	NA	.0086	1.6373	-	NA	.0271
(1,R3)	.0000	NA	.0000	NA	-	NA
(2,R3)	NA	.0000	NA	.000	.0006	-

Supplementary Figure 3. Phylogenetic tree excerpted from Supplementary Fig. 1. Caesalpinioideae subtree showing enumerated gains and losses of RNS (Supplementary Tables 1 and 3). Some speciose clades are scaled, collapsed, and labeled to allow for easier viewing.

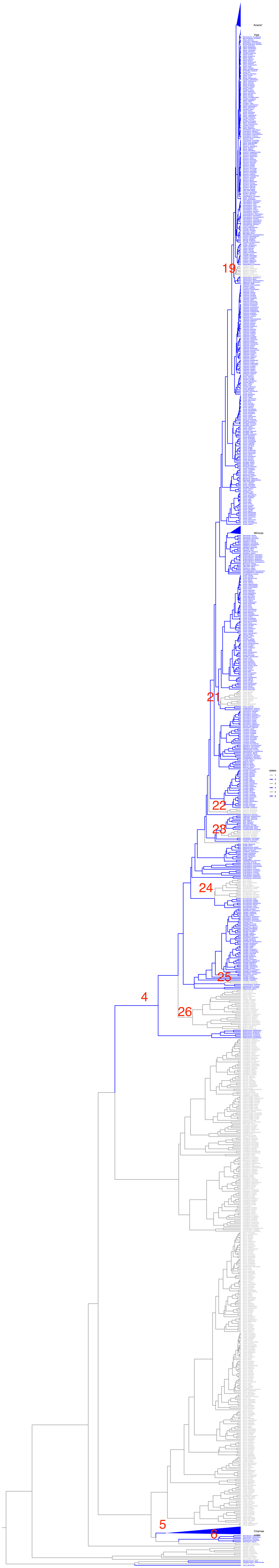

Acacia*
Inga
19
Mimosa
21
states
1
2
3
4
5
6
22
23
24
25
4
26
5
6
Chamae
crista

Supplementary Figure 4. Phylogenetic tree excerpted from Supplementary Fig. 1. Rosales subtree showing enumerated gains and losses of RNS (Supplementary Tables 1 and 3). Some speciose clades are scaled, collapsed, and labeled to allow for easier viewing.

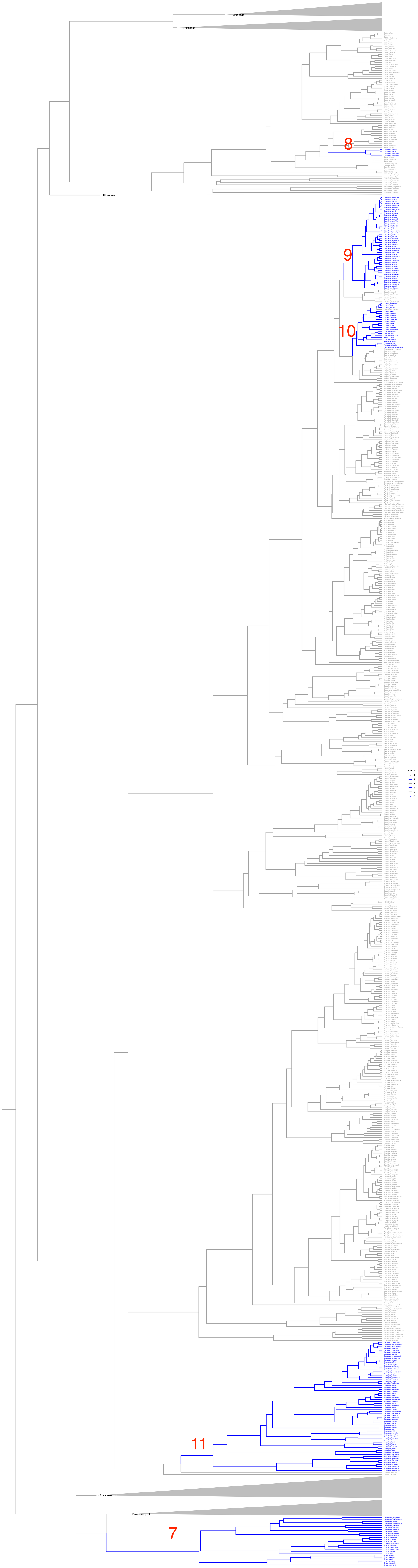
Moraceae
Urticaceae
8
Ulmaceae
9
10
states
11
Rosaceae pt. 2
Rosaceae pt. 1
7

Supplementary Figure 5. Phylogenetic tree excerpted from Supplementary Fig. 1. Fagales subtree showing enumerated gains and losses of RNS (Supplementary Tables 1 and 3). Some speciose clades are scaled, collapsed, and labeled to allow for easier viewing.

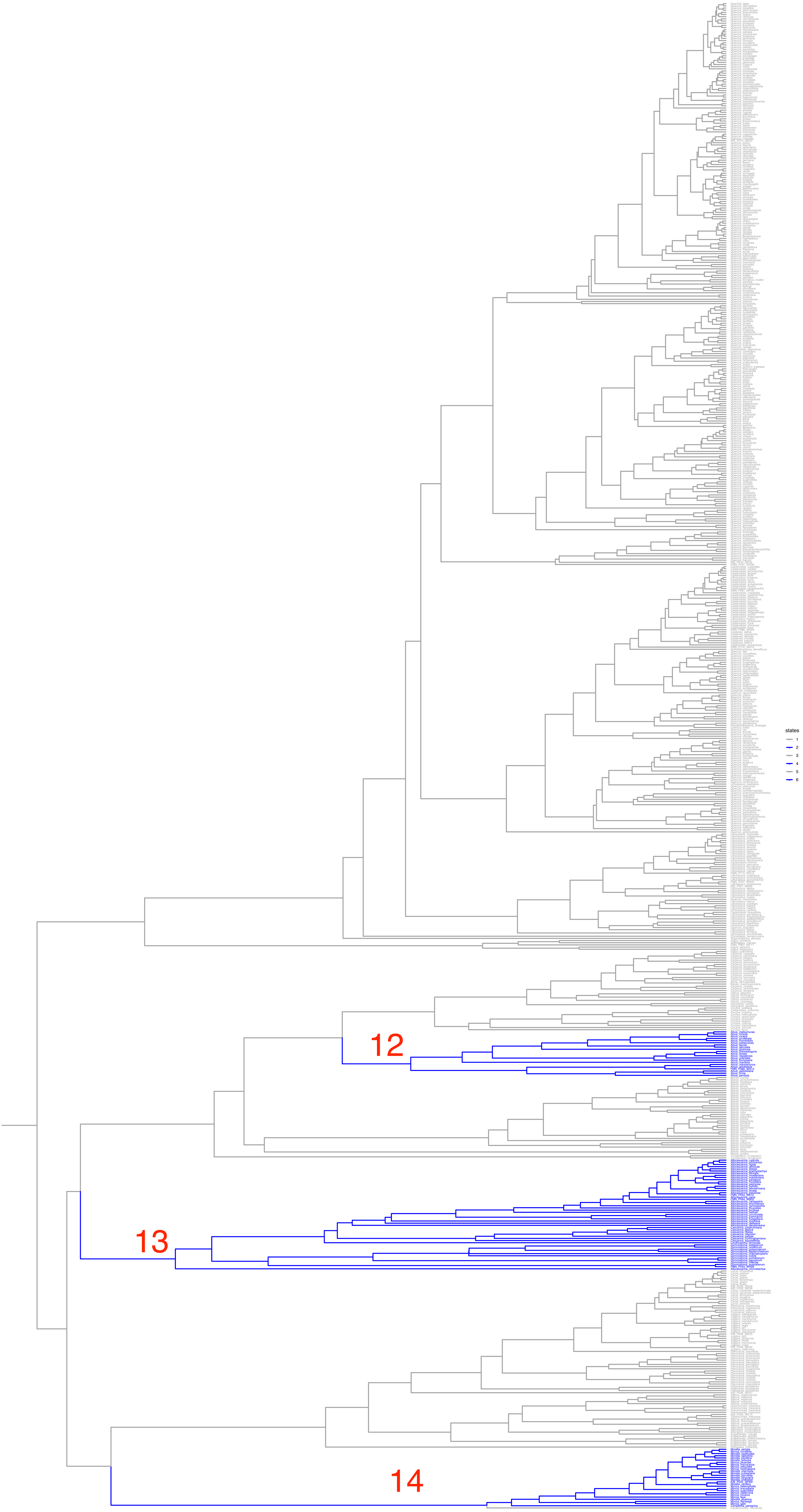
states
1
2
3
4
5
6
12
13
14

Supplementary Figure 6. Phylogenetic tree excerpted from Supplementary Fig. 1. Cucurbitales subtree to show enumerated gains and losses of RNS (Supplementary Tables 1 and 3). Some speciose clades are scaled, collapsed, and labeled to allow for easier viewing.

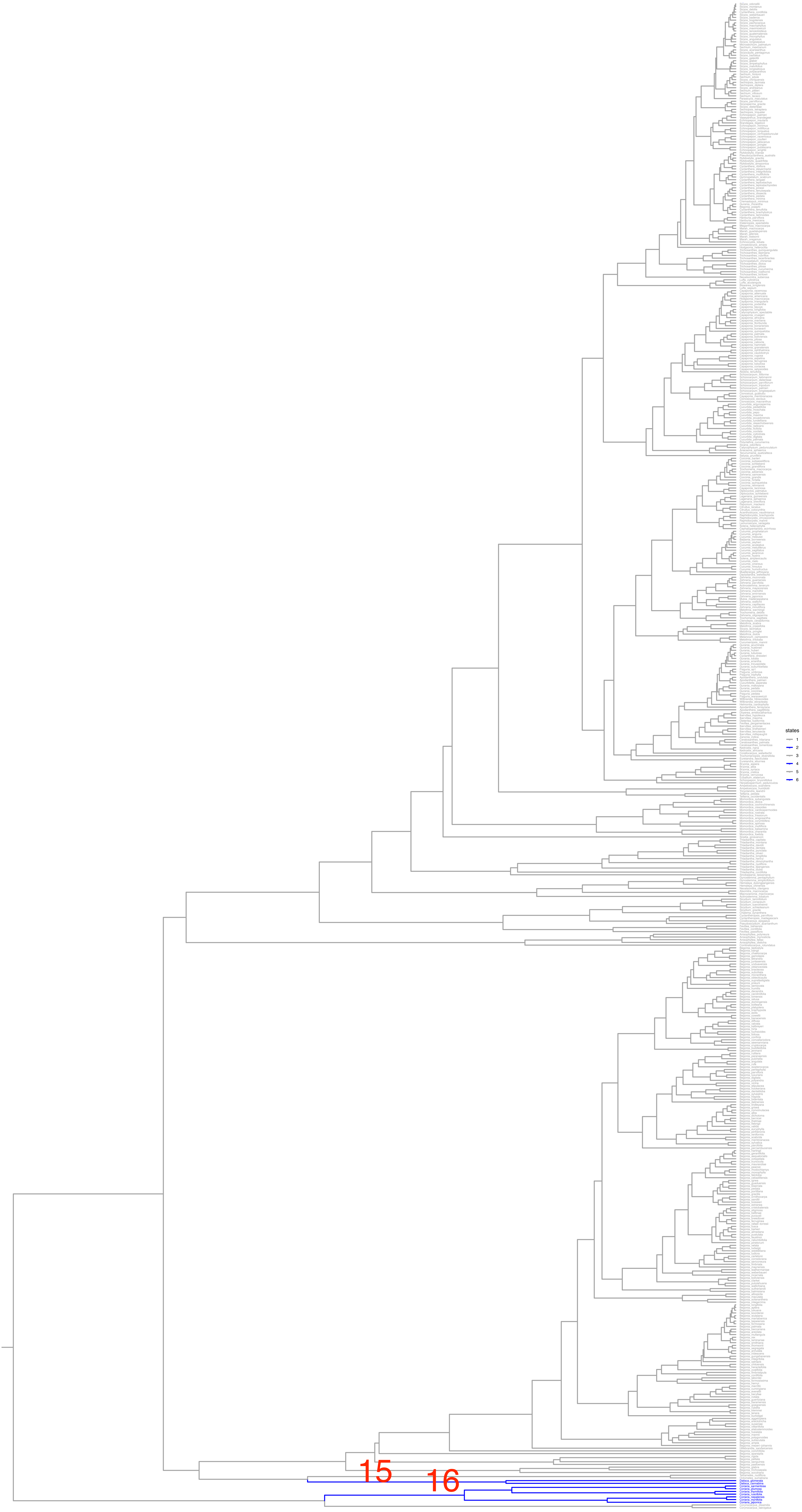
states
1
2
3
4
5
6
15
16

Supplementary Figure 7. Phylogenetic tree excerpted from Supplementary Fig. 1. Papilionoideae subtree showing enumerated gains and losses of RNS (Supplementary Tables 1 and 3). Some speciose clades are scaled, collapsed, and labeled to allow for easier viewing.

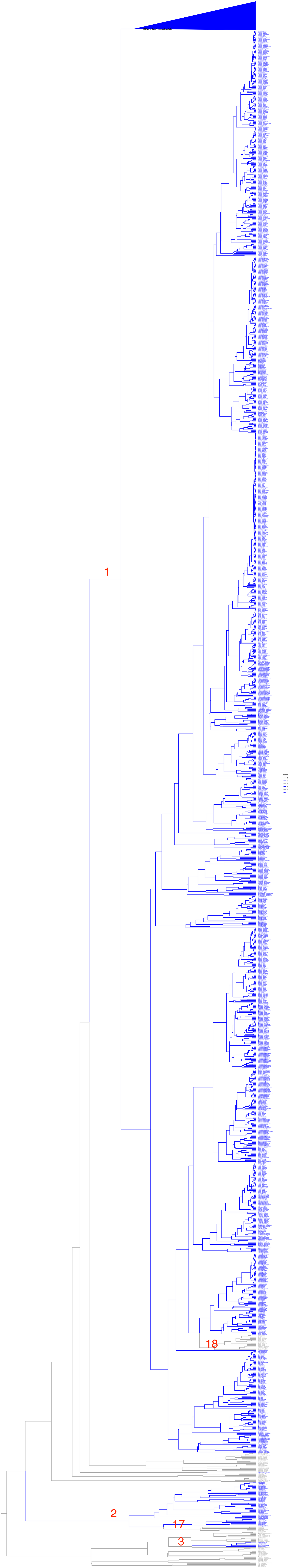
1
18
2
17
3

Supplementary Figure 8. Results of a set of simulated transition rates for a two-rate model of RNS gain and loss. Gain rate is on the X-axis, and loss rate is on the Y-axis. Plot background is colored by logLikihood scores for the plotted resulting model likelihoods; worse likelihoods are at the bottom, and better likelihoods are at the top. For each model, if the resulting marginal reconstruction estimated the NFC ancestor as RNS-absent, the plot point is colored red. (Ancestral RNS-presence would result in blue plot points, but no models yielded this result.) Circle at bottom right indicates the upper limit of the rate of loss tested (when loss of nodulation is over 60 times more likely than gain) at which a marginal character state reconstruction estimates that the ancestor of the NFC lacks RNS.

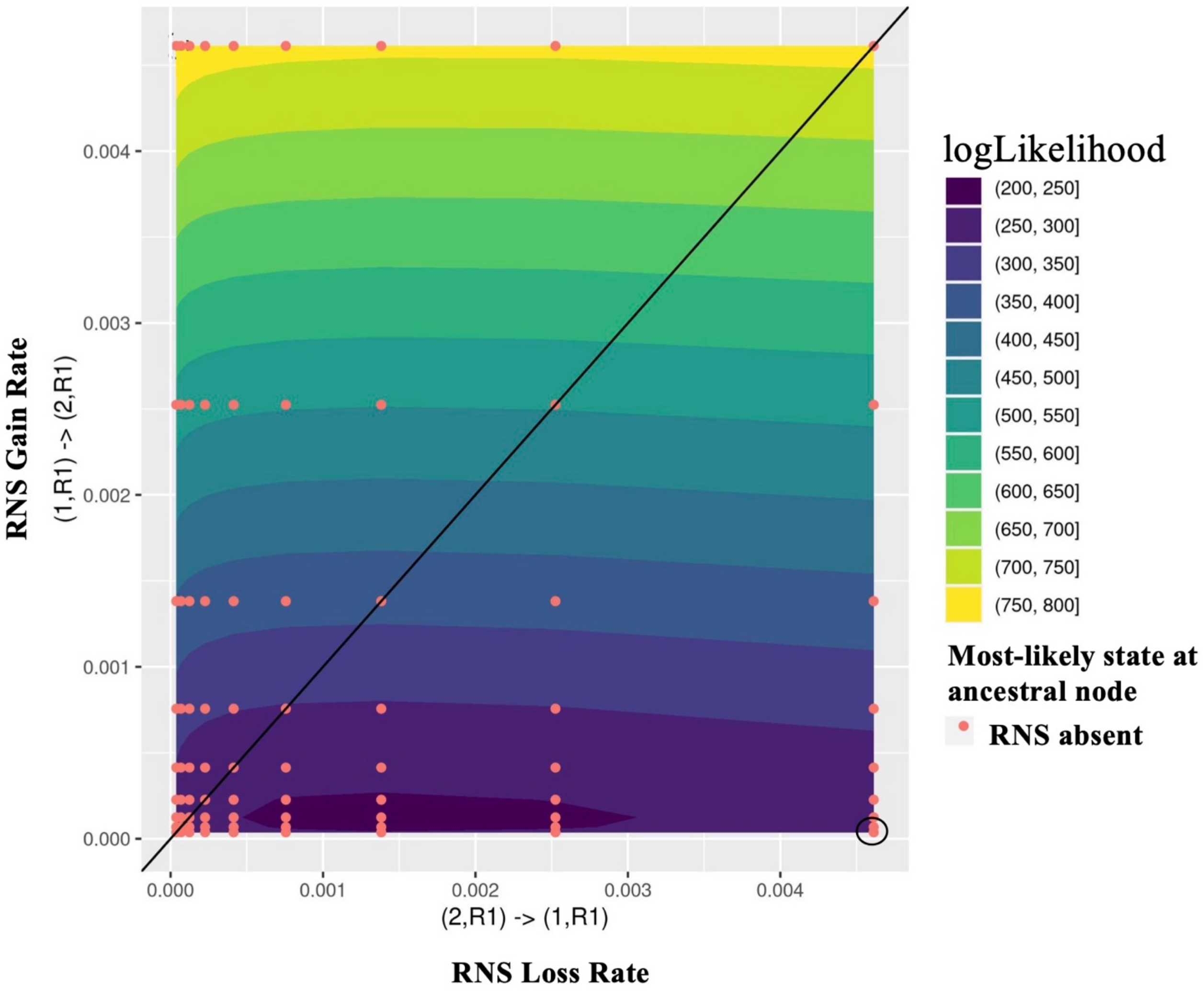

Supplementary Figure 9. Results of transition rate estimation and ancestral character state reconstruction in fixed-ancestral-state (RNS-present) analysis. A. Character states inferred by joint estimation of ancestral character states. Non-NFC clades are scaled down (unlabeled section) to highlight the NFC. NFC orders and legume subfamilies are indicated by colored bars. Branches are colored by the state estimated at their child node. B. A linear representation of the NFC phylogeny to highlight transitions in basal nodes that are not readily visible in the circular format; the taxon sequence is identical to that in the circle tree. C. Transition rate matrix of estimated rates from states listed on left to states listed at top. Rows are colored to correspond to colors in panel A.

A

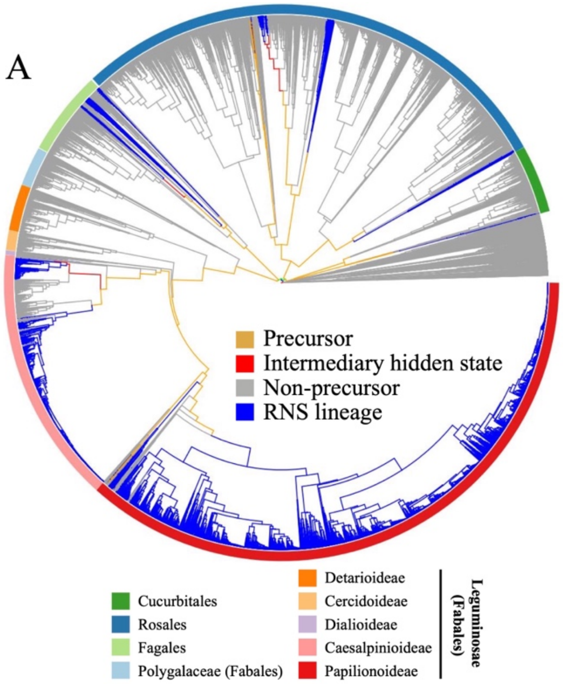

B

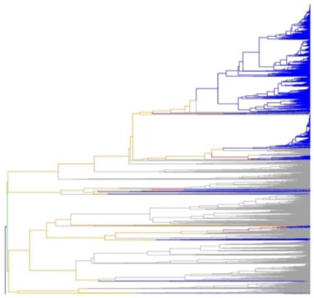

C

	(1,R1)	(2,R1)	(1,R2)	(2,R2)	(1,R3)	(2,R3)
(1,R1)	-	.000	.000	NA	.000	NA
(2,R1)	.001	-	NA	.000	NA	.000
(1,R2)	.109	NA	-	.100	.022	NA
(2,R2)	NA	.109	.000	-	NA	.022
(1,R3)	.028	NA	.008	NA	-	NA
(2,R3)	NA	.028	NA	.008	.797	-

Supplementary Figure 10. Supplementary phylogenetic trees. Twenty subsampled backbone phylogenies are presented based on ten sets of representative subsamples: Pages 1-10 were inferred using ASTRAL (page 3 is the backbone phylogeny used for subtree scaffolding and the main phylogeny presented in this paper). Pages 11-20 were inferred using IQ-tree.

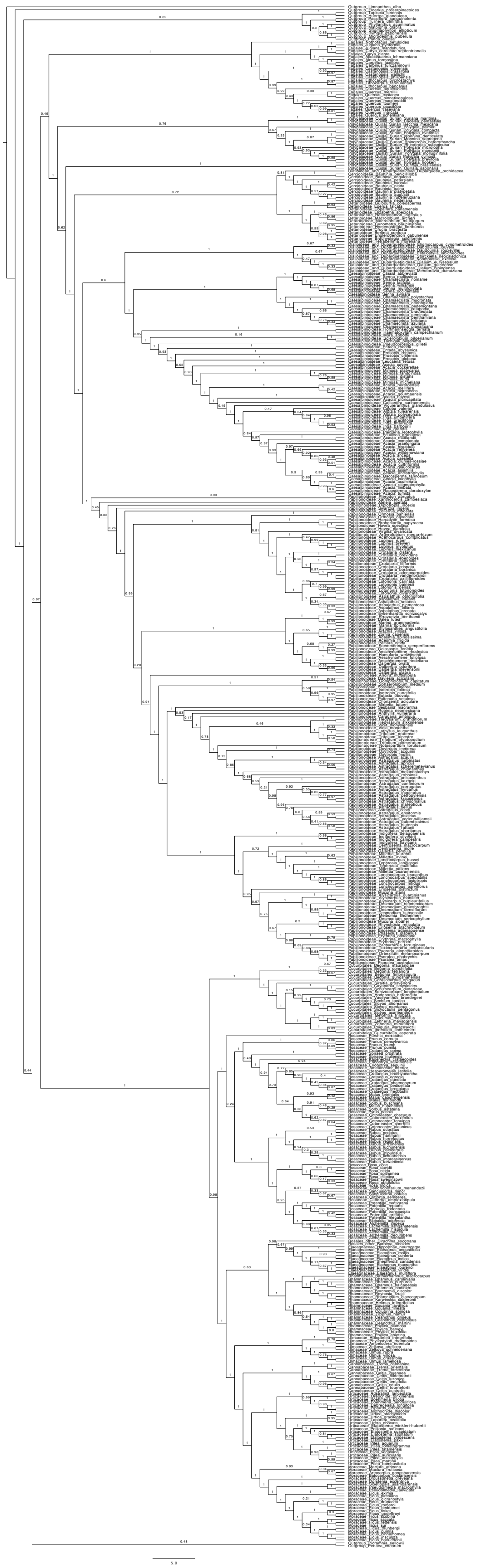
5.0

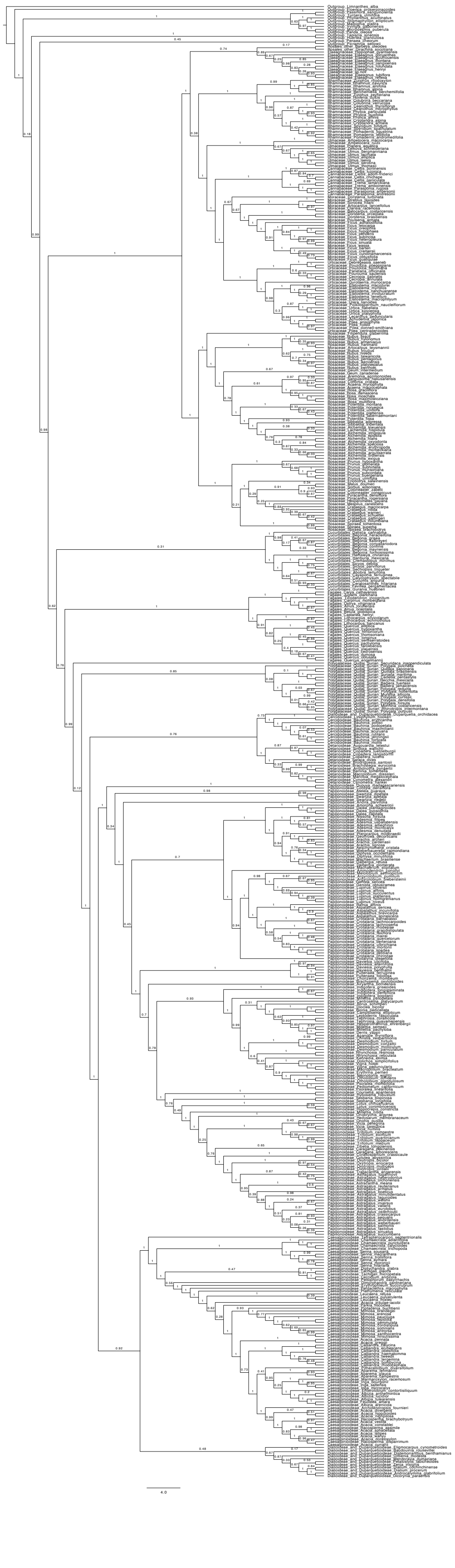

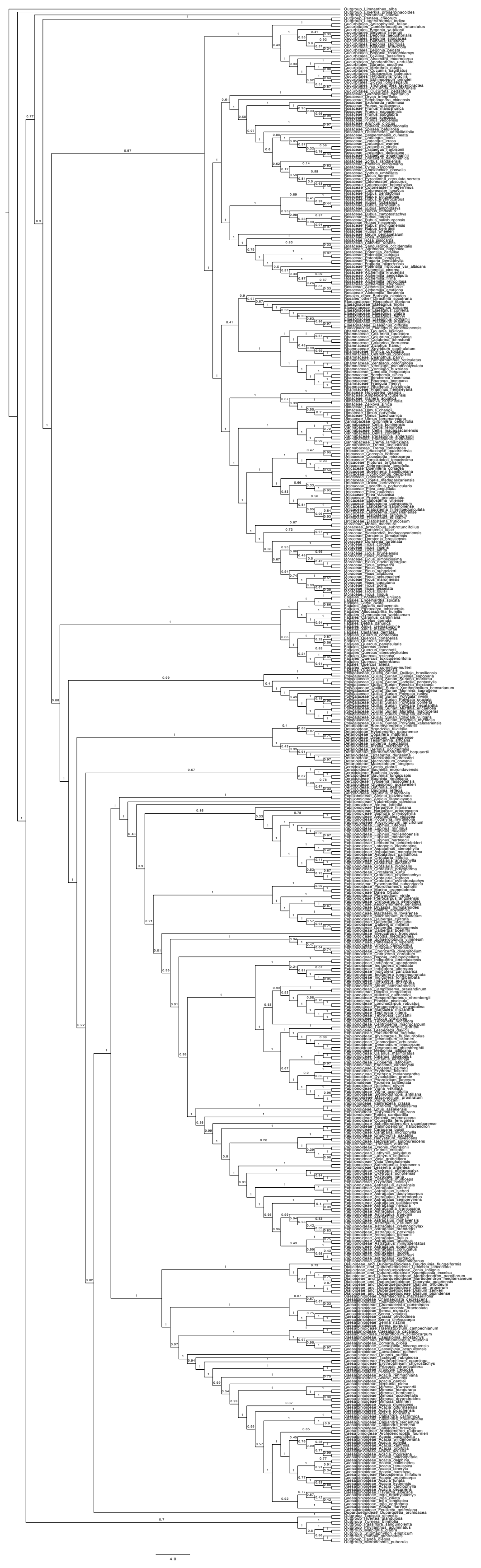

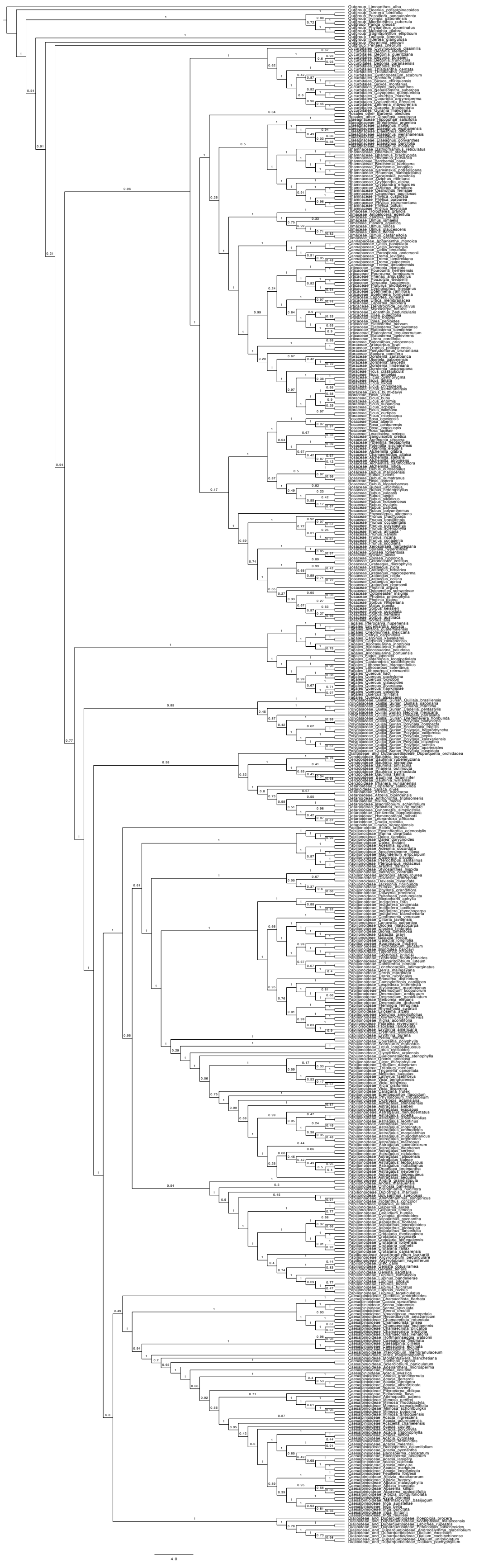

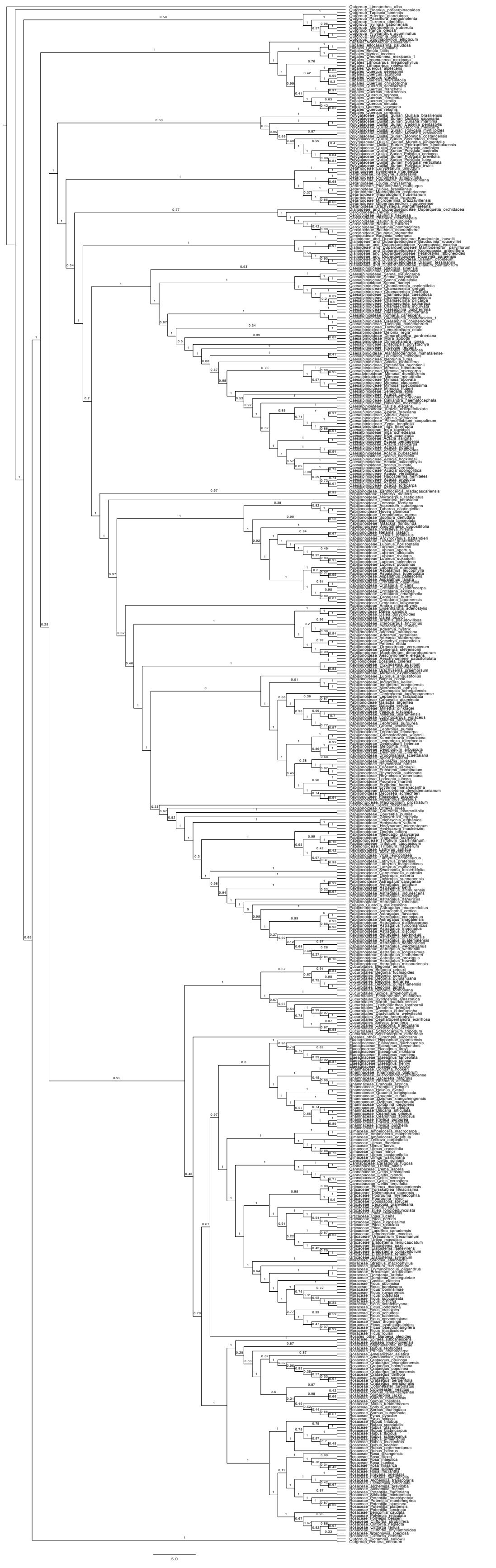

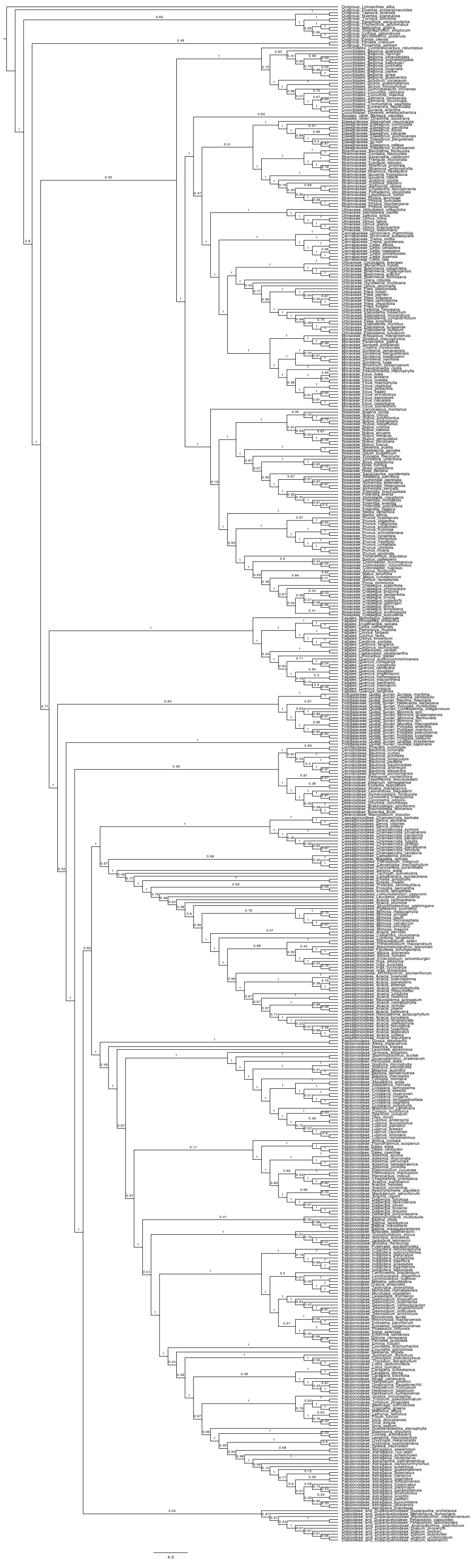

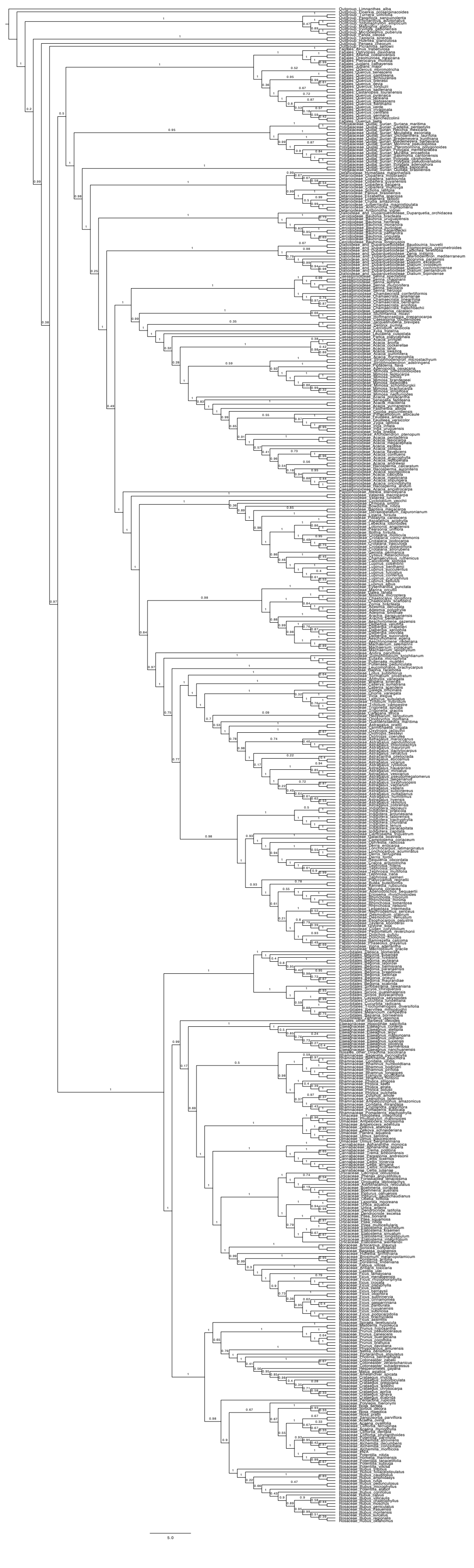

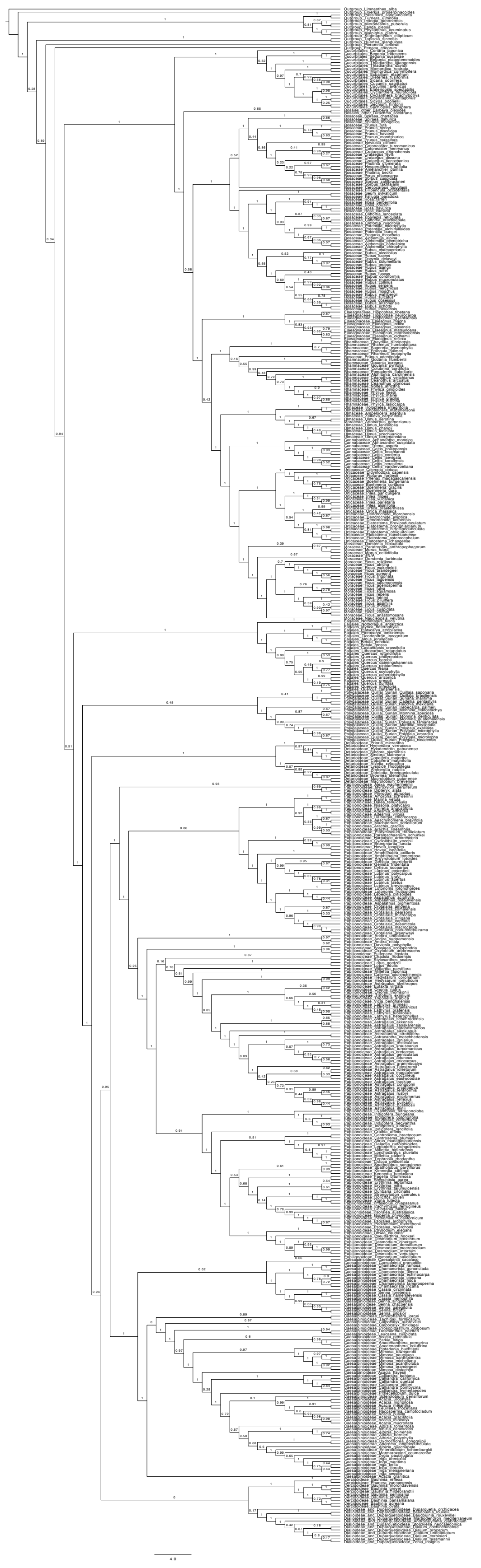

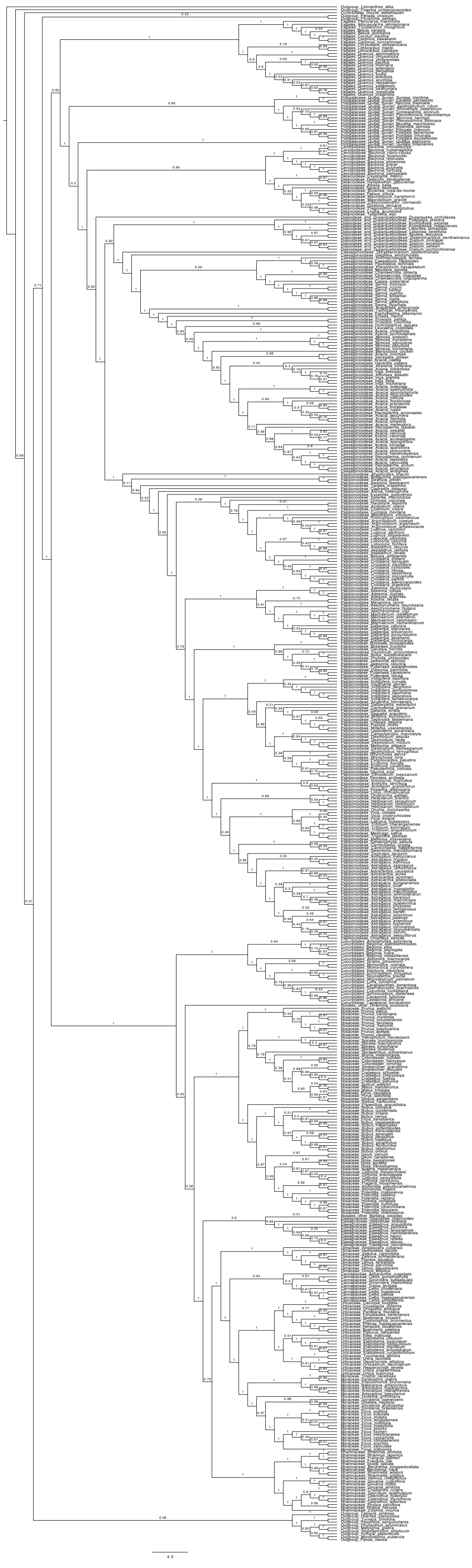

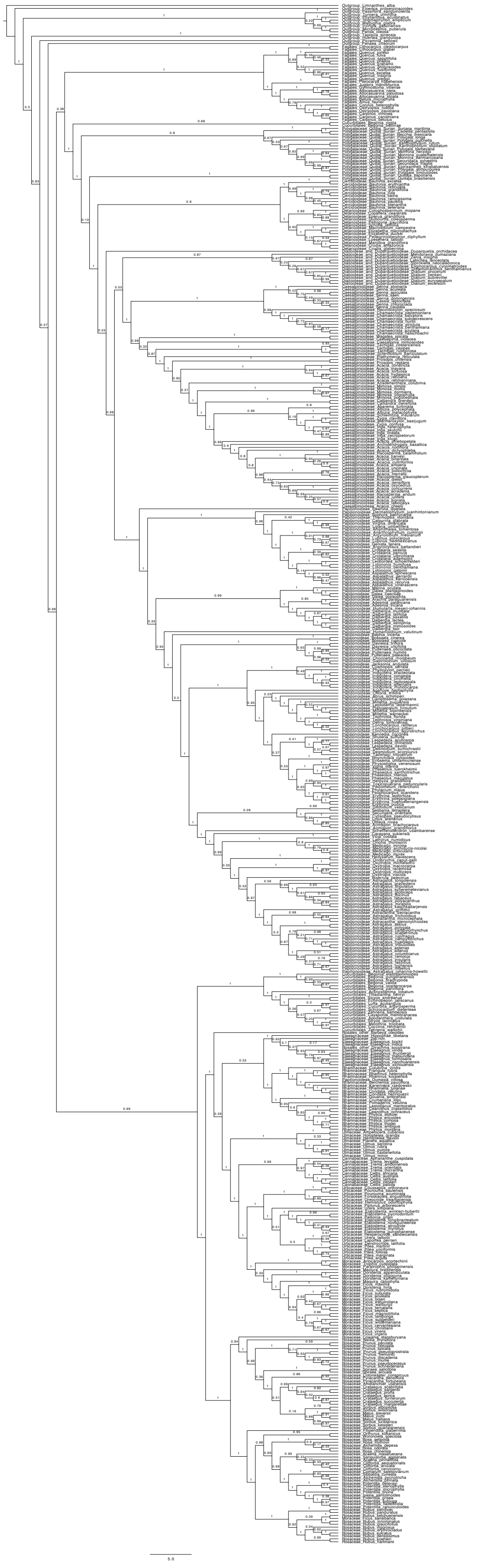

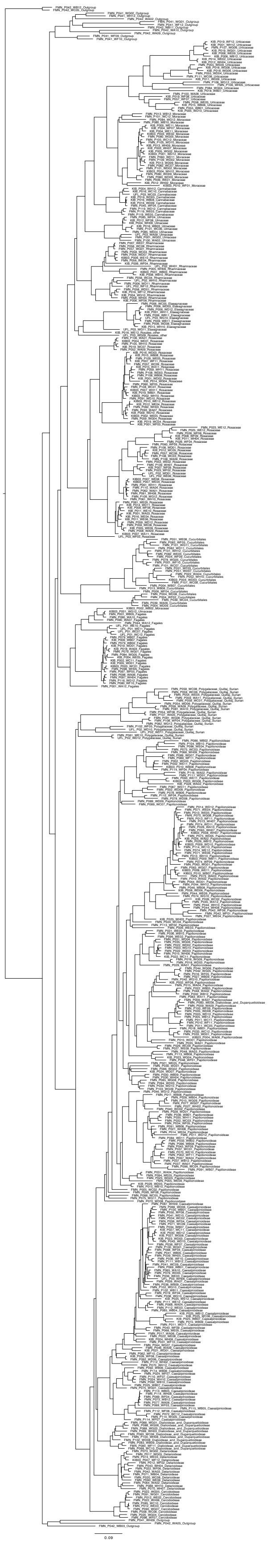
0.09

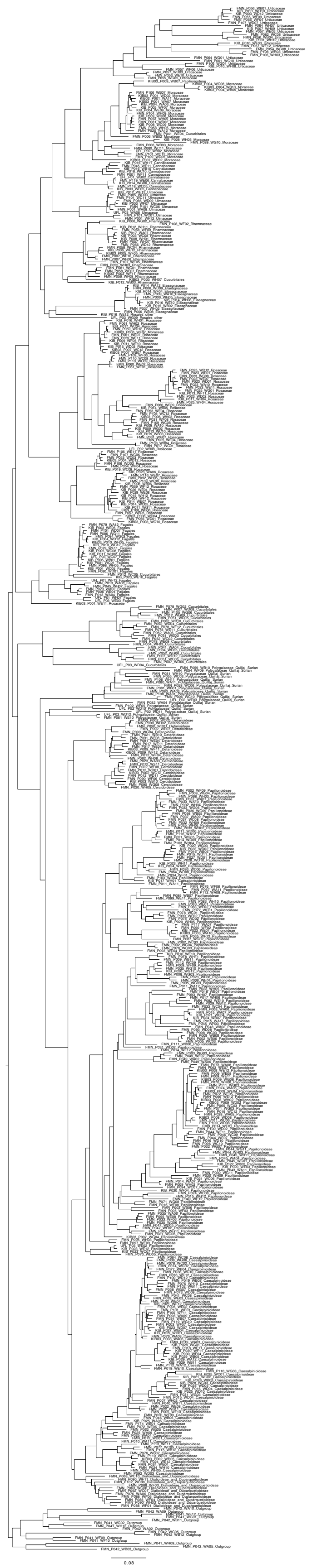
0.08

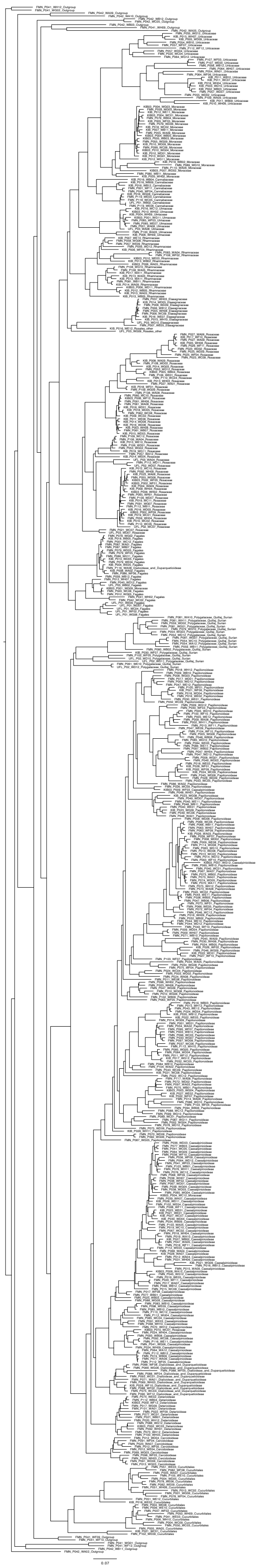
0.07

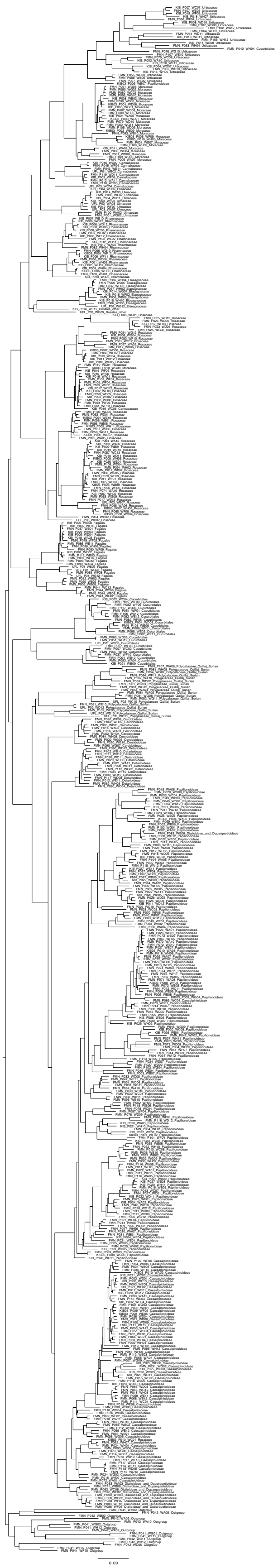
0.09

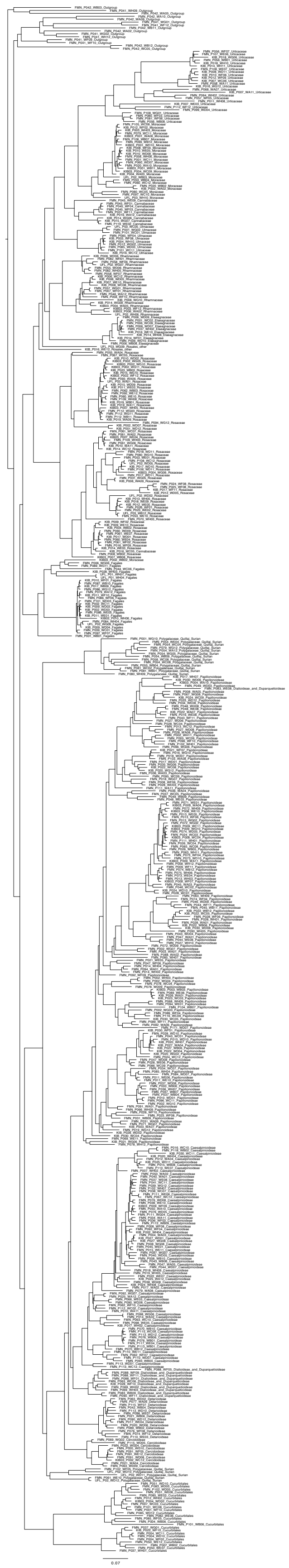

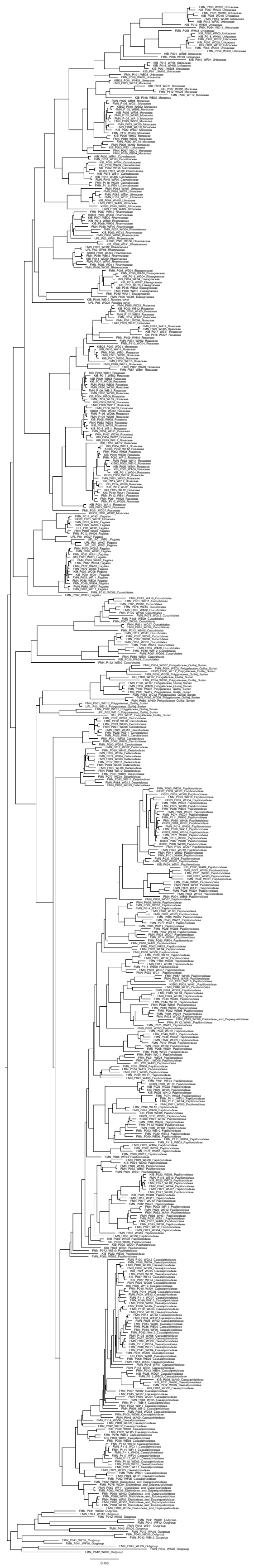
0.09

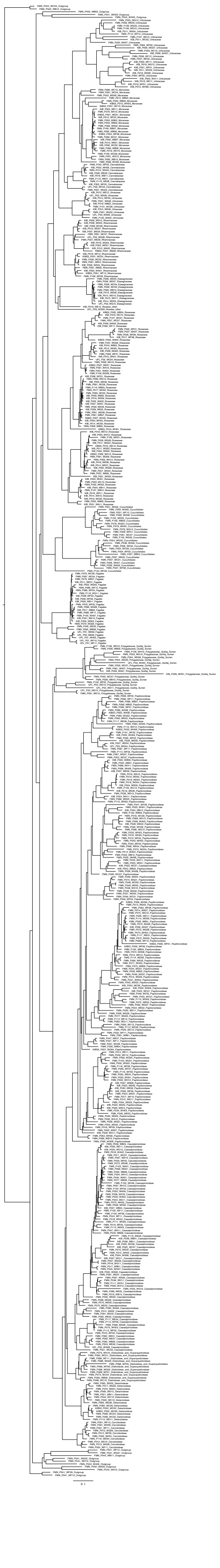

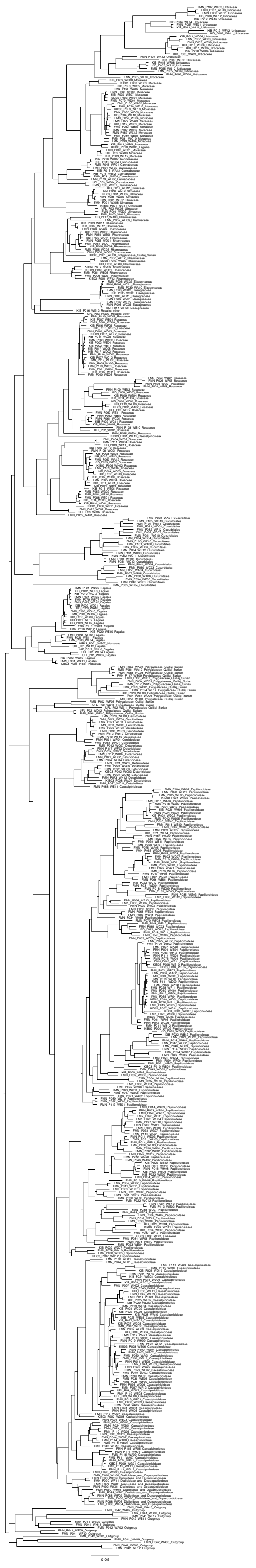
0.08

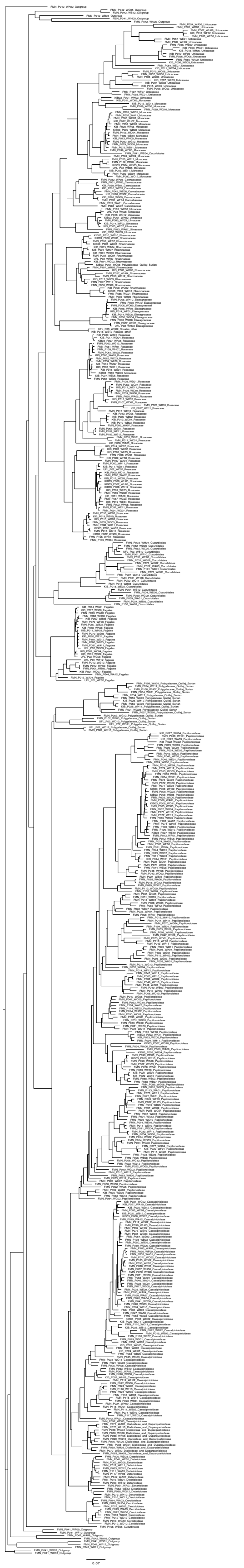

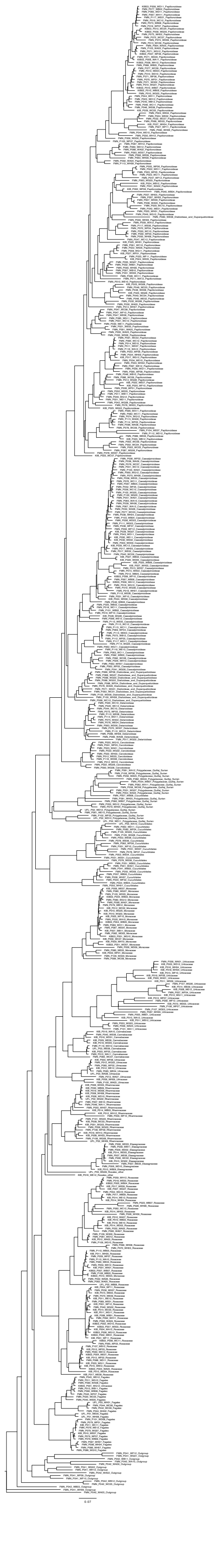

Supplementary Figure 11. Results of transition rate estimation and ancestral character state reconstruction based on the two-rate precursor model (deltaAIC 1.063). A. Character states inferred by joint estimation of ancestral character states. Non-NFC clades are scaled down (unlabeled section) to highlight the NFC. NFC orders and legume subfamilies are indicated by colored bars. Branches are colored by the state estimated at their tipward node. B. Transition rate matrix of estimated rates from states listed on left to states listed at top. Rows are colored to correspond to colors in panel A.

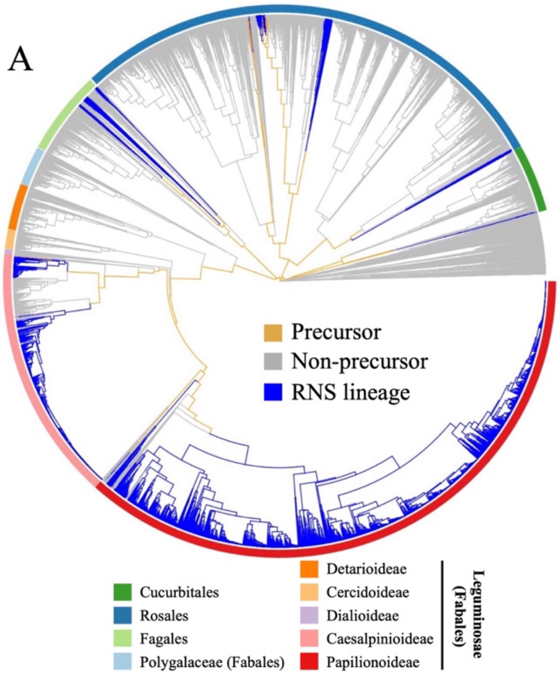

B

	(1,R1)	(2,R1)	(1,R2)	(2,R2)
(1,R1)	-	.0045	.0369	NA
(2,R1)	.0079	-	NA	.0369
(1,R2)	.0000	NA	-	NA
(2,R2)	NA	.0000	.0006	-

Supplementary Figure 12. Results of transition rate estimation and ancestral character state reconstruction based on a model of ordered RNS-state change with three states (RNS-absent, Actinorhizal-RNS, and Rhizobial-RNS) and two rate categories (delta AIC 49.176). A. Character states inferred by joint estimation of ancestral character states. Non-NFC clades are scaled down (unlabeled section) to highlight the NFC. NFC orders and legume subfamilies are indicated by colored bars. Branches are colored by the state estimated at their tipward node. B. Transition rate matrix of estimated rates from states listed on left to states listed at top. Rows are colored to correspond to colors in panel A.

A

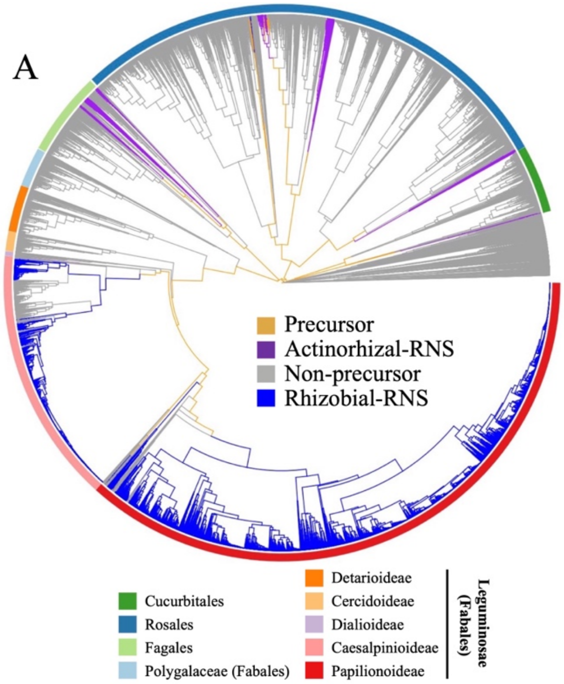

B

	(1,R1)	(2,R1)	(3,R1)	(1,R2)	(2,R2)	(3,R2)
(1,R1)	-	.0045	NA	.0374	NA	NA
(2,R1)	.0643	-	.0235	NA	.0374	NA
(3,R1)	.0134	NA	-	NA	NA	.0374
(1,R2)	.0000	NA	NA	-	NA	NA
(2,R2)	NA	.0000	NA	.0001	-	.0000
(3,R2)	NA	NA	.0000	.0007	NA	-

Supplementary Figure 13. Network of transition rates estimated by the R package corHMM (see Methods) showing transitions from and to hidden states. Each hidden state is an observed state (RNS-present or RNS-absent) in one of three rate categories. State circle and transition arrow colors correspond with trait mapping on the phylogeny in Supplementary Fig. 1. Arrows show transitions between states and are scaled proportionally to the rate at which the transition occurs, except for the transition 2,R2→1,R2, which is 30% of actual width to allow for visualization of the smallest rates, and 2,R3→1,R3, which is 15x actual width to increase visibility. Transition rates are displayed below arrows or to the left in four cases where arrows were oriented vertically. Numeric rates and 95% confidence-intervals are reported in Supplementary Table 3.

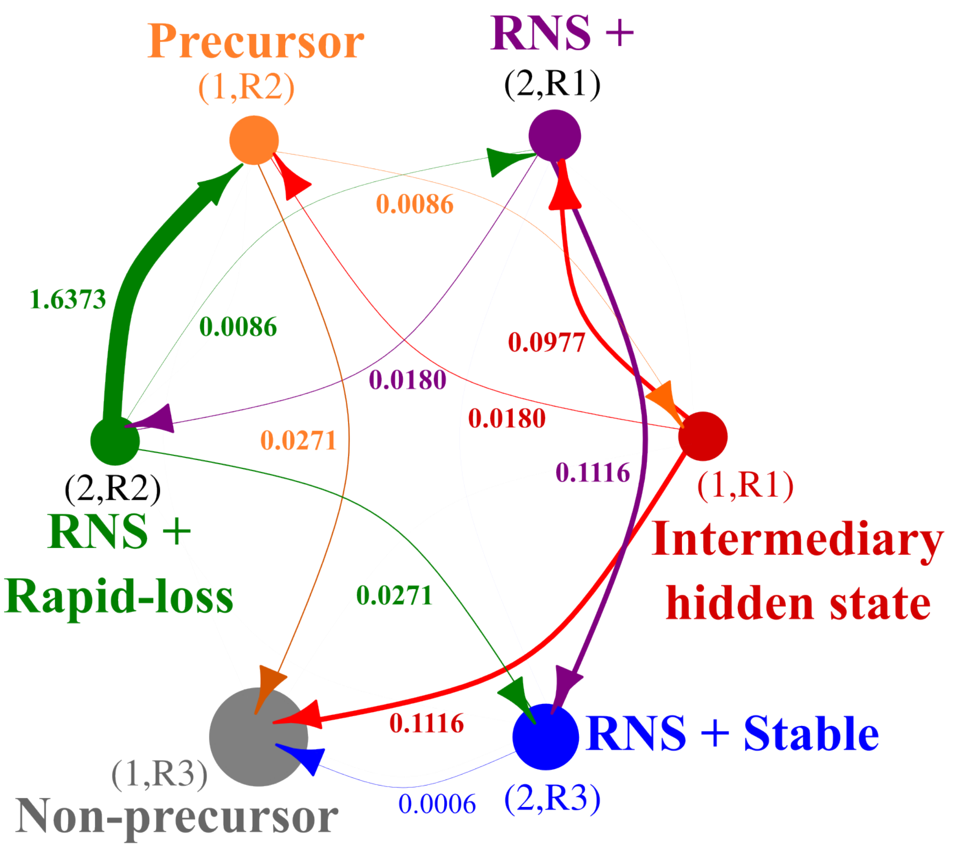

Supplementary Note 1. Summary of NFC topology resolved in this study

The relationships we resolved among the four NFC orders (Cucurbitales, Fabales, Fagales, and Rosales) are congruent with recent multilocus nuclear-gene based phylogenies[1,2] that differ from earlier trees based primarily on the chloroplast genome that have often been used to analyze or discuss the evolution of SNF[3,4]. Some within-order relationships in our tree are novel but strongly supported (Supplementary Data 2, p. 3). For example, Caesalpinioideae and Papilionoideae, the two predominantly nodulating and the most speciose clades in the NFC, are typically sister[5], but we find instead that Caesalpinioideae is sister to the non-RNS subfamily Dialioideae. The topology used for the evolutionary models represents our current best evidence, but these relationships may be difficult to definitively resolve; for example, there was likely a near-simultaneous evolutionary origin of all six legume subfamilies[2].

Supplementary Note 2. Detail on single-copy target-locus filtering

Although we designed probes to target putatively single-copy loci, the phylogenetic breadth of the dataset, which includes ancient and recent polyploidy (e.g. Leguminosae[6,7]; Rosaceae[8]; Cucurbitaceae[9]), means that no locus is single-copy across all samples, as demonstrated by the aTRAM assembly. When multiple copies of a target gene are present in a dataset, the ideal strategy for assessing orthology would be to include all paralogs in the phylogenetic analysis. Because this was not feasible at the phylogenetic and data scales considered here, we instead designed an alternative subclade approach using phylogenetic information to reduce the likelihood of erroneously analyzing paralogs while minimizing data loss, as described below.

We performed single-copy target-locus filtering for each subclade and four outgroup samples independently (15 major clades: Fagales, Cucurbitales, seven Rosales families, one non-Leguminosae Fabales family, and five Leguminosae subfamilies; five additional clades with few species were analyzed with a closely related larger clade). For each clade, *de novo* contigs were first assembled using SPAdes[10] as implemented in the target-locus assembly pipeline aTRAM2[11]. We built and used a custom aTRAM function called atram_framer.py to put the *de novo* contigs into reading frame based on the target-locus reference sequence and filtered by coverage using a 40X coverage cutoff. We then used scripts from Yang and Smith[12] to build a phylogeny of contig

sequences and identify all samples that had contigs that were not sister in the contig phylogeny; these contigs were dropped from the target-locus dataset for that clade. In cases where multiple contigs that were assembled for a single sample were sister in the phylogeny we retained the contig with the longest sequence length. Contigs that passed coverage (> 40X) and length (> 250 bp) cutoffs and were present in single copy in the contig tree were carried on to target-locus alignment and phylogenetic analysis. Scripts are available at the GitHub repository ().

Supplementary Note 3. Detail on RNS-state database and genera that were coded as unknown in the analysis

Nine legume genera could not be reasonably scored as RNS-present or RNS-absent because no information was available from either published observations or expert input (Supplementary Table 1) and because their phylogenetic positions in hotspots of RNS evolution did not allow for reasonably confident inference of RNS state. Species from these genera were scored as unknown in the transition rate estimation, and their states were inferred by the joint reconstruction analysis. In five cases where the inferred state would represent an independent loss or gain of RNS, we did not include these in our reported results in extended data tables 2 and 3. In Caesalpinioideae, *Arcoa* and *Tetrapterocarpon* are both inferred by ancestral character state reconstruction as RNS-present, requiring two independent gains, and *Sympetalandra* is inferred as lacking nodules, requiring an independent loss. In Papilionoideae, *Amphimas* is inferred as having nodules present, requiring an independent gain. There are unconfirmed field reports of nodulation in *Amphimas*[13] that were met with skepticism by some experts[14], but given the present evidence, it is possible that this represents a true gain of RNS. *Petaladenium* is inferred as lacking nodules, requiring a loss.

Inferences of nodulation state for the remaining genera with unknown RNS data had no effect on inferred gains or losses: *Viguieranthus* is inferred as present but because of its phylogenetic position its inference in either state has no effect on gains or losses. However, because of its sister relationship to a genus that exhibits an independent loss, *Zapoteca*, confirmation of nodulation status in this genus is a high priority. *Diptychandra* is inferred as present, but does not require an additional gain. *Dussia* is inferred as present, as preliminarily reported by Saur et al.[15];

because of its phylogenetic position, its inference in either state has no effect on reconstruction of gains or losses.

In Caesalpinioideae, the non-monophyly of two genera, *Mora* (RNS-absent) and *Dimorphandra* (RNS-present), known previously[16], precluded our genus-level scoring of RNS. To resolve this issue, the following *Dimorphandra* species were coded as lacking nodules based on phylogenetic position and no species-level observations: *Dimorphandra cuprea*, *D. ignea, D. vernicosa, D. pennigera,* and *D. polyandra*, as shown below.

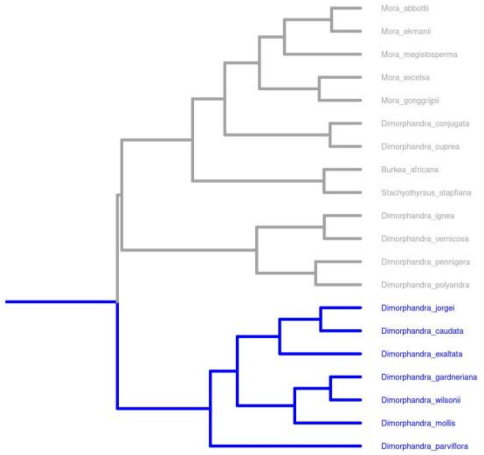

Supplementary Note 4. Results of character state reconstruction using an alternative backbone topology of the NFC

The relationships among the four orders of the NFC and among legume subfamilies are uncertain due to rapid divergences of these clades in evolutionary history. To check how alternative backbone topologies besides our best-evidence result affect the reconstructed history of RNS, we merged subtrees for these orders and subfamilies together onto a backbone representing relationships commonly recovered in previous studies and used to study RNS. Relationships among orders were set according to APG I[17], i.e., (((Fagales,Rosales),Cucurbitales),Fabales), largely representing chloroplast phylogenetic information, and relationships among legume subfamilies were set according to Koenen et al[2]. The analysis of Koenen et al.[2] omitted the legume subfamily Duparquetioideae and the Fabales family Surianaceae; we inserted these in positions congruent with our main phylogeny. We reran our hidden state estimation and

reconstruction on this phylogeny using identical methods. The overall result (Supplementary Fig. 3A) is highly consistent with the reconstruction using the best-evidence topology recovered with our data: the ancestor of the NFC is in the deep-precursor state, and the same number of independent origins of SNF is hypothesized (Extended Data Table 2). One notable difference is that the direct-precursor state is not recovered at such a high rate (compare Figure 2 and Supplementary Fig. 3B), meaning the reconstructed state is not as transient and therefore is seen more often in the character state reconstruction.

References

1. Zhao, L. *et al.* Phylogenomic analyses of large-scale nuclear genes provide new insights into the evolutionary relationships within the rosids. *Mol. Phylogenet. Evol.*, **105,** 166-176, (2016).
2. Koenen, E. J. M. *et al.* Large-scale genomic sequence data resolve the deepest divergences in the legume phylogeny and support a near-simultaneous evolutionary origin of all six subfamilies. *New Phytol.* **225**, 1355–1369 (2020).
3. van Velzen, R., Doyle, J. J. & Geurts, R. A Resurrected Scenario: Single Gain and Massive Loss of Nitrogen-Fixing Nodulation. *Trends Plant Sci.* **24**, 49–57 (2019).
4. Doyle, J. J. Chasing unicorns: Nodulation origins and the paradox of novelty. *Am J Bot.* **103**, 1865-1868, doi: 10.3732/ajb.1600260 (2016).
5. Legume Phylogeny Working Group (LPWG). A new subfamily classification of the Leguminosae based on a taxonomically comprehensive phylogeny. *Taxon*, **66**, 44-77. https://doi.org/10.12705/661.3 (2017).
6. Wojciechowski, M.F. (2019), Tracing the history of chromosome evolution in legumes using genomics. New Phytol, 223: 1693-1695. https://doi.org/10.1111/nph.15926
7. Cannon S. B., *et al.* Multiple polyploidy events in the early radiation of nodulating and nonnodulating legumes. *Mol Biol Evol.* **1**, 193-210. doi: 10.1093/molbev/msu296. (2014).
8. Xiang Y., et al.., Evolution of Rosaceae fruit types based on nuclear phylogeny in the context of geological times and genome duplication. *Mol. Biol. Evol.* **34**, 262–281 (2017).
9. Jing Guo *et al.* Phylotranscriptomics in Cucurbitaceae Reveal Multiple Whole-Genome Duplications and Key Morphological and Molecular Innovations, *Molecular Plant*, **13**, 1117-1133 (2020).

10. Bankevich A, *et al.* SPAdes: a new genome assembly algorithm and its applications to single-cell sequencing. *J Comput Biol.* **19**, 455-77. doi: 10.1089/cmb.2012.0021 (2012).
11. Allen, J. M., LaFrance, R., Folk, R. A., Johnson, K. P. & Guralnick, R. P. aTRAM 2.0: An Improved, Flexible Locus Assembler for NGS Data. *Evol. Bioinform. Online* **14**, 1176934318774546 (2018).
12. Yang, Y. & Smith, S. A. Orthology inference in nonmodel organisms using transcriptomes and low-coverage genomes: improving accuracy and matrix occupancy for phylogenomics. *Mol. Biol. Evol.* **31**, 3081–3092 (2014).
13. Diabate, M. *et al.* Occurrence of nodulation in unexplored leguminous trees native to the West African tropical rainforest and inoculation response of native species useful in reforestation. *New Phytol.* **166**, 231–239 (2005).
14. Sprent, J. West African legumes: the role of nodulation and nitrogen fixation. *New Phytologist*, **167**, 326-330. https://doi.org/10.1111/j.1469-8137.2005.01499.x (2005).
15. Saur, E., Carcelle, S., Guezennec, S. & Rousteau, A. Nodulation of legume species in wetlands of guadeloupe (lesser antilles). *Wetlands*, **20**, 730–734 (2000).
16. The Legume Phylogeny Working Group, Legume phylogeny and classification in the 21st century: Progress, prospects and lessons for other species–rich clades. *Taxon*, **62**, 217-248. https://doi.org/10.12705/622.8 (2013)
17. The Angiosperm Phylogeny Group. An update of the Angiosperm Phylogeny Group classification for the orders and families of flowering plants: APG IV, *Botanical Journal of the Linnean Society*, **181**, 1–20, https://doi.org/10.1111/boj.12385 (2016).
