# Supplementary material for: Shifts in evolutionary lability underlie independent gains and losses of root-nodule symbiosis in a single clade of plants
Source: Nat Commun. 2024 May 27;15:4262. doi: 10.1038/s41467-024-48036-3 (PMC11130336; doi:10.1038/s41467-024-48036-3)
Supplement: Supplementary file 3 — Description of Additional Supplementary Files - NEW [file 41467_2024_48036_MOESM3_ESM.pdf]

Description of Additional Supplementary Files:

Supplementary Data 1: Samples used in study. Taxon names in tree differ from scientificName_Voucher when taxonomy has been updates per internally shared file from Kew/Legume Phylogeny Working Group.

Supplementary Data 2: RNS-state trait database.

Supplementary Data 3: Phylogenetic tree presented in Supplementary Data 1 in newick format. Branches are colored by all six inferred hidden state (ancestral RNS status + rate category). For full explanation of colors and rate categories, see Supplementary Figure 13: RNS+ =blue ("stable"), purple (no name), and green ("rapid-loss"); RNS-absent=orange ("precursor"), red ("intermediary hidden-state", and gray ("non-precursor").
