# Supplementary material for: Shifts in evolutionary lability underlie independent gains and losses of root-nodule symbiosis in a single clade of plants
Source: Nat Commun. 2024 May 27;15:4262. doi: 10.1038/s41467-024-48036-3 (PMC11130336; doi:10.1038/s41467-024-48036-3)
Supplement: Supplementary file 7 — Reporting Summary [file 41467_2024_48036_MOESM7_ESM.pdf]

nature portfolio

Corresponding author(s): Heather R. Kates and Ryan A. Folk

Last updated by author(s): Feb 9, 2024

Reporting Summary

Nature Portfolio wishes to improve the reproducibility of the work that we publish. This form provides structure for consistency and transparency in reporting. For further information on Nature Portfolio policies, see our Editorial Policies and the Editorial Policy Checklist.

Statistics

For all statistical analyses, confirm that the following items are present in the figure legend, table legend, main text, or Methods section.

n/a	Confirmed	
x		The exact sample size (*n*) for each experimental group/condition, given as a discrete number and unit of measurement
x		A statement on whether measurements were taken from distinct samples or whether the same sample was measured repeatedly
	x	The statistical test(s) used AND whether they are one- or two-sided<br>*Only common tests should be described solely by name; describe more complex techniques in the Methods section.*
x		A description of all covariates tested
	x	A description of any assumptions or corrections, such as tests of normality and adjustment for multiple comparisons
	x	A full description of the statistical parameters including central tendency (e.g. means) or other basic estimates (e.g. regression coefficient) AND variation (e.g. standard deviation) or associated estimates of uncertainty (e.g. confidence intervals)
	x	For null hypothesis testing, the test statistic (e.g. *F*, *t*, *r*) with confidence intervals, effect sizes, degrees of freedom and *P* value noted<br>*Give P values as exact values whenever suitable.*
x		For Bayesian analysis, information on the choice of priors and Markov chain Monte Carlo settings
x		For hierarchical and complex designs, identification of the appropriate level for tests and full reporting of outcomes
x		Estimates of effect sizes (e.g. Cohen's *d*, Pearson's *r*), indicating how they were calculated

Our web collection on statistics for biologists contains articles on many of the points above.

Software and code

Policy information about availability of computer code

Data collection	n/a
Data analysis	Specimen management: SLIMS v0.1.1 (https://github.com/rafelafrance/nitfix). Sequencing panel design: MarkerMiner v1. Sequence QC: FastQC v0.11.9, Trimmomatic v0.39. Assembly: aTRAM v2. Alignment: MAFFT v7.294b. Phylogenetic inference: IQ-tree v1.6.12, ASTRAL vIII, RAxML-NG v1.1.0. Time calibration: treePL v1.087.

For manuscripts utilizing custom algorithms or software that are central to the research but not yet described in published literature, software must be made available to editors and reviewers. We strongly encourage code deposition in a community repository (e.g. GitHub). See the Nature Portfolio guidelines for submitting code & software for further information.

Data

Policy information about availability of data

All manuscripts must include a data availability statement. This statement should provide the following information, where applicable:

- Accession codes, unique identifiers, or web links for publicly available datasets
- A description of any restrictions on data availability
- For clinical datasets or third party data, please ensure that the statement adheres to our policy

Supporting Information is available online in Supplemental Information and Data, including RNS state database, phylogenetic trees, and detailed plots of ancestral reconstruction. Sequence data are available on

SRA (BioProject PRJNA1021556 (https://www.ncbi.nlm.nih.gov/bioproject/1021556), PRJNA1021608 (https://www.ncbi.nlm.nih.gov/bioproject/1021608), PRJNA1021620 (https://www.ncbi.nlm.nih.gov/bioproject/1021620), PRJNA1022015 (https://www.ncbi.nlm.nih.gov/bioproject/1022015), PRJNA1022023 (https://www.ncbi.nlm.nih.gov/bioproject/1022023), PRJNA1022025 (https://www.ncbi.nlm.nih.gov/bioproject/1022025), PRJNA1022027 (https://www.ncbi.nlm.nih.gov/bioproject/1022027), PRJNA1022029 (https://www.ncbi.nlm.nih.gov/bioproject/1022029), PRJNA1022030 (https://www.ncbi.nlm.nih.gov/bioproject/1022030), PRJNA1022032 (https://www.ncbi.nlm.nih.gov/bioproject/1022032), PRJNA1022323 (https://www.ncbi.nlm.nih.gov/bioproject/1022323), PRJNA1022138 (https://www.ncbi.nlm.nih.gov/bioproject/1022138), PRJNA1022141 (https://www.ncbi.nlm.nih.gov/bioproject/1022141), PRJNA1022147 ((https://www.ncbi.nlm.nih.gov/bioproject/1022147)), and the phylogenetic tree from Fig. 1 and Supplementary Fig. 1 is
available in OpenTree (Study ID ot_2291; https://tree.opentreeoflife.org/curator/study/view/ot_2291) and in newick format as Supplementary Data File 3. A concatenated alignment file including all samples (used for branch length estimation and can be parsed into smaller subsets for subtree analyses) is Supplementary Data File 4.

Research involving human participants, their data, or biological material

Policy information about studies with human participants or human data. See also policy information about sex, gender (identity/presentation), and sexual orientation and race, ethnicity and racism.

Reporting on sex and gender	n/a
Reporting on race, ethnicity, or other socially relevant groupings	n/a
Population characteristics	n/a
Recruitment	n/a
Ethics oversight	n/a

Note that full information on the approval of the study protocol must also be provided in the manuscript.

Field-specific reporting

Please select the one below that is the best fit for your research. If you are not sure, read the appropriate sections before making your selection.

☐ Life sciences ☐ Behavioural & social sciences ☒ Ecological, evolutionary & environmental sciences

For a reference copy of the document with all sections, see nature.com/documents/nr-reporting-summary-flat.pdf

Ecological, evolutionary & environmental sciences study design

All studies must disclose on these points even when the disclosure is negative.

Study description	Large-scale study using expansive phylogenomic data and a comprehensive trait database representing nodulation states to infer ancestral nodulation states and the rate of nodule evolution.
Research sample	12,768 species of the nitrogen-fixing clade (NFC), a monophyletic clade of flowering plants. The samples were incorporated into a previously published study covering a larger clade known as the rosids (Sun, M., R.A. Folk, M.A. Gitzendanner, P.S. Soltis, Z. Chen, D.E. Soltis, and R.P. Guralnick. 2020. Recent, accelerated diversification in rosids occurred outside the tropics. Nature Communications 11: 3333. https://doi.org/10.1038/s41467- 020-17116-5) as described exhaustively in the main text.
Sampling strategy	Sampling was phylogenetically representative, with partially automated sampling decisions guided using a comprehensive taxonomic resource (The Plant List v1.1), information from the nodulation dataset developed in the current study, and consultation of the primary literature.
Data collection	Data were collected through destructive sampling of herbarium specimens, as described in the main text. The primary participants were (among the authors) Ryan A. Folk, Daniel Conde, Robert P. Guralnick, Heather R. Kates, Douglas E. Soltis, Pamela S. Soltis, Tingshuang Yi, Shuiyin Liu, Qin Tian, Tingshuang Yi, Matias Kirst, and the individuals mentioned in the acknowledgments section. The method of data collection has been reported extensively in a published manuscript (Folk, R.A., H.R. Kates, R. LaFrance, D.E. Soltis, P.S. Soltis, and R.P. Guralnick. 2021. High-throughput methods for efficiently building massive phylogenies from natural history collections. Applications in Plant Sciences 9: e11410.).
Timing and spatial scale	Timing of sample collection: November 2017 - July 2018. Timing of sequencing: May 2018 - . Spatial scale: Global.
Data exclusions	Raw data were excluded on the basis of a priori criteria, which were either of (1) extremely low DNA yield (< 10 ng), (2) failed sequencing or (3) detected cross-contamination, which was based on BLAST searches against GenBank's NR database. 14 of the 100 loci sequenced in total were excluded across all samples due to high levels of paralogy using methods described in the main text.
Reproducibility	Maximum likelihood modeling was conducted in 100 replicates to ensure that the global maximum likelihood was found.

Randomization	The backbone phylogeny was inferred using a randomization procedure involving random sampling of 500 species in 20 replicates. Our study did not involve experimental groups so no randomization of this type was used.
Blinding	Our study did not involve experimental groups so experimental blinding was not relevant.

Did the study involve field work? ☐ Yes ☒ No

Reporting for specific materials, systems and methods

We require information from authors about some types of materials, experimental systems and methods used in many studies. Here, indicate whether each material, system or method listed is relevant to your study. If you are not sure if a list item applies to your research, read the appropriate section before selecting a response.

Materials & experimental systems

n/a	Involved in the study	
☒	☐	Antibodies
☒	☐	Eukaryotic cell lines
☒	☐	Palaeontology and archaeology
☒	☐	Animals and other organisms
☒	☐	Clinical data
☒	☐	Dual use research of concern
☐	☒	Plants

Methods

n/a	Involved in the study	
☒	☐	ChIP-seq
☒	☐	Flow cytometry
☒	☐	MRI-based neuroimaging

Plants

Seed stocks	Plant tissue from herbarium specimens.
Novel plant genotypes	n/a
Authentication	n/a
